# The Ocean Carbon and Acidification Data System

**DOI:** 10.1038/s41597-023-02042-0

**Published:** 2023-03-15

**Authors:** Li-Qing Jiang, Alex Kozyr, John M. Relph, Errol I. Ronje, Linus Kamb, Eugene Burger, Jonathan Myer, Liem Nguyen, Krisa M. Arzayus, Tim Boyer, Scott Cross, Hernan Garcia, Patrick Hogan, Kirsten Larsen, A. Rost Parsons

**Affiliations:** 1 Cooperative Institute for Satellite Earth System Studies, Earth System Science Interdisciplinary Center, University of Maryland, College Park, Maryland 20740 USA; 2 NOAA/NESDIS National Centers for Environmental Information, Silver Spring, Maryland 20910 USA; 3 NOAA/NESDIS National Centers for Environmental Information, Stennis Space Center, Mississippi 39529 USA; 4 NOAA/OAR Pacific Marine Environmental Laboratory, Seattle, Washington 98115 USA; 5 NOAA/NESDIS National Centers for Environmental Information, Asheville, North Carolina 28801 USA; 6 Department of Computer Science, University of Maryland, College Park, Maryland 20740 USA; 7 NOAA/NOS Integrated Ocean Observing System, Silver Spring, Maryland 20910 USA; 8 NOAA/NESDIS National Centers for Environmental Information, Charleston, South Carolina 29412 USA

**Keywords:** Ocean sciences, Biogeochemistry, Climate sciences

## Abstract

The Ocean Carbon and Acidification Data System (OCADS) is a data management system at the National Oceanic and Atmospheric Administration (NOAA) National Centers for Environmental Information (NCEI). It manages a wide range of ocean carbon and acidification data, including chemical, physical, and biological observations collected from research vessels, ships of opportunity, and uncrewed platforms, as well as laboratory experiment results, and model outputs. Additionally, OCADS serves as a repository for related Global Ocean Observing System (GOOS) biogeochemistry Essential Ocean Variables (EOVs), e.g., oxygen, nutrients, transient tracers, and stable isotopes. OCADS endeavors to be one of the world’s leading providers of ocean carbon and acidification data, information, products, and services. To provide the best data management services to the ocean carbon and acidification research community, OCADS prioritizes adopting a customer-centric approach and gathering knowledge and expertise from the research community to improve its data management practices. OCADS aims to make all ocean carbon and acidification data accessible via a single portal, and welcomes submissions from around the world: https://www.ncei.noaa.gov/products/ocean-carbon-acidification-data-system/.

## Introduction

With ~71% of its surface area covered by the ocean, the Earth is sometimes called a “Blue Planet” or “Water Planet”. The ocean plays a critical role in climate regulation by sequestering and storing approximately 25% of anthropogenic carbon dioxide (CO_2_), one of the primary greenhouse gases emitted by human activities^1–6^. Furthermore, the ocean absorbs about 90% of the excess heat trapped in the Earth system and transports heat from the equator to the poles^7,8^. Additionally, the ocean provides various ecosystem goods and services that are essential to our society. Billions of people, especially those living in the coastal communities, depend on the ocean for their food security^9,10^, recreation^11^, livelihood^12,13^, and natural and cultural heritage^14^.

Since the beginning of the Industrial Revolution around 1750, human activities have released ~2.5 trillion metric tons of carbon dioxide^1–6^, resulting in an atmospheric CO_2_ increase of ~50% (~419 parts per million, or ppm, 2022 annual average)^15^ compared to the 1750 level of ~277 ppm^16^, causing our climate to change^17,18^. Without the carbon capture and sequestration services provided by the ocean, the atmospheric CO_2_ today would have been ~80 ppm higher than the current level^3^.

This large influx of carbon dioxide is altering the ocean’s chemistry. After CO_2_ is absorbed by seawater, a portion of it will react with water to form carbonic acid, which will then dissociate and release hydrogen ions (H^+^). The extra H^+^ will associate with carbonate ions (CO_3_^2−^) to increase the concentration of bicarbonate ions (HCO_3_^−^). The net result is that the mildly alkaline ocean is becoming more acidic (and less alkaline), and its carbonate ion (CO_3_^2−^), a building block for many marine organisms, has been decreasing. This process is commonly referred to as “ocean acidification (OA)”^19–26^. Ocean acidification is making it harder for marine calcifiers (e.g., mollusks, crustaceans, coral) to build and maintain their shells and skeletal structures, endangering coral reefs and marine ecosystems more broadly^27–30^. It can also negatively affect non-calcifying organisms, e.g., disrupting the use of chemical sensations to find food or avoid predators for certain species, thus potentially shifting the food web, and changing the community composition and structure^31,32^. Even apex predators (e.g., large whales) may experience the effects of ocean acidification in the future as decreasing pH levels could change the acoustic characteristics of the ocean, thus alter or disrupt their communication and foraging behaviors as the ocean becomes noisier^33–35^.

On timescales of decades to millennia, the ocean imposes a dominant control over atmospheric CO_2_ levels, due to its vast size and efficient exchange of CO_2_ with the atmosphere^5,36,37^. It contains the majority (~95%) of the total active pool of inorganic carbon in the Earth’s surface, at ~38,000 billion tons of carbon, which is ~45 times that of the atmosphere^38–40^. Geoengineering efforts, such as marine carbon dioxide removal (mCDR), could potentially change the ocean’s chemistry and impact the marine ecosystems^41^. Therefore, understanding carbon cycling in the ocean is critical for the research of global climate change, ocean acidification, marine CDR, and their downstream effects on the marine ecosystems, and the final impact on the human socio-economic structure^28,42^.

**Fig. 1:**
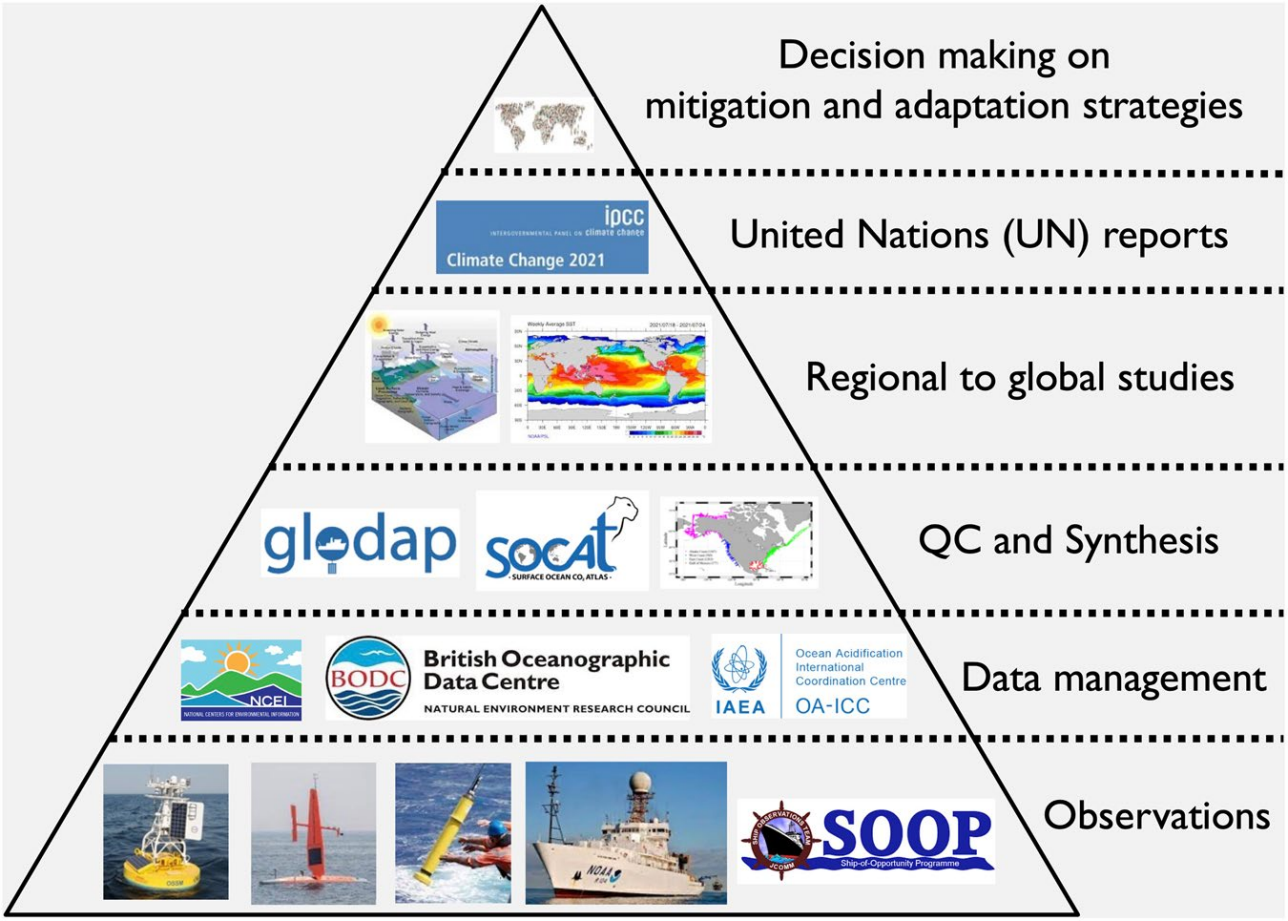
A schematic diagram showing the importance of ocean data management in promoting oceanographic research and product developments. (Credit: This figure is adapted from a diagram generated by Guidi *et al*.^58^).

Effective data management is a crucial aspect of the research efforts mentioned above^43,44^ (Fig. 1). After all, the ocean is a global system, and data collected from individual research cruises often need to be compiled into regional and global data products, before they can be used to support further oceanographic research, enable the Measurement, Reporting, and Verification (MRV) of mCDR for carbon credit accounting, and produce reports that can guide society’s strategies for mitigating and adapting to environmental changes^45^. Data management provides an avenue where all data are (a) safeguarded for long-term access; (b) documented with common metadata and data standards and controlled vocabularies; and (c) findable and accessible. It plays a very important role in data reuse and valorization, model verification, and most importantly, quality control (QC), synthesis, and data product developments.

The Ocean component of the Carbon Dioxide Information Analysis Center (CDIAC-Oceans, Oak Ridge National Laboratory, Oak Ridge, Tennessee, USA) played a very important role in the management of ocean carbon data from 1993 to 2016. Two factors contributed to the success of CDIAC-Oceans: its customer-centric approach, and the accessibility of all ocean carbon data collected by the global research community through a single data portal. However, CDIAC-Oceans’ data holdings were not backed with a long-term archive. Its portfolio was focused on ocean carbon data and related hydrographic variables only. Its infrastructure was not designed to manage other types of OA data, such as physiological response biological studies. Worst of all, CDIAC-Oceans lost its funding from the Department of Energy in 2016.

NCEI’s OA data management efforts started with an NOAA Ocean Acidification Program (OAP) funded project called Ocean Acidification Data Stewardship (OADS) in 2012. By leveraging a modern community-driven rich metadata template^46^ and NCEI’s enterprise long-term archive management system with version control and controlled vocabulary management capabilities, OADS built state-of-the-art technical infrastructure that helped transform NCEI’s OA data management efforts from a top-down government task to a bottom-up community-driven service.

In November 2015, NCEI was notified that CDIAC-Oceans would lose its funding by the end of September 2016. To continue the service that was provided by CDIAC-Oceans, a new project called Ocean Carbon Data System (OCADS) was established at NCEI in January 2017, using the same technical infrastructure that was built by OADS. All CDIAC’s data holdings and webpages were transferred to NCEI, and the CDIAC-Oceans staff member was hired by NCEI as a cooperative institute employee to continue his work on the management of international ocean carbon data. The Ocean Carbon Data System could be considered as CDIAC-Oceans 2.0. All data holdings were upgraded with a much-improved modern metadata template^46^. Controlled vocabularies were applied to all aspects of the metadata to ensure accurate data findability. All data files were published into NCEI’s long-term archive with version control capabilities.

In September 2022, a new integrated system called the Ocean Carbon and Acidification Data System (OCADS) was formed at NCEI out of a merger of the former Ocean Acidification Data Stewardship (OADS) and Ocean Carbon Data System (OCADS). This merger was a response to feedback from the research community, who had encountered difficulties in choosing between the two systems for submitting and accessing data purposes. With the merger, the entire suite of ocean carbon and acidification data services offered by NCEI became available to the global research community, ushering in a new era of collaborative research possibilities. It’s worth noting that the acronym OCADS remains the same, even though the new OCADS is a consolidation of the two previous systems.

Besides NCEI, current data management systems that have ocean carbon or acidification data in their portfolios include the CLIVAR and Carbon Hydrographic Data Office (CCHDO, USA), Biological and Chemical Oceanography Data Management Office (BCO-DMO, USA), the Ocean acidification International Coordination Center (OA-ICC, Monaco), PANGAEA (Germany), European Marine Observation and Data Network (EMODnet, Belgium), British Oceanographic Data Center (BODC, U.K.), Bjerknes Climate Data Centre (Norway), International Oceanographic Data and Information Exchange (IODE) Sustainable Development Goals (SDG) Portal (France), OceanOPS (France), and Joint European Research Infrastructure for Coastal Observatories (JERICO).

The Ocean Carbon and Acidification Data System has been the choice of data management services for most of the recent ocean carbon and acidification data product developments, e.g., the Surface Ocean CO_2_ Atlas Version 2022 (SOCATv2022)^47^, Global Ocean Data Analysis Project  Version 2 (GLODAPv2.2022)^48^, the Coastal Ocean Data Analysis Product in North America (CODAP-NA)^49^, and the CARbon, tracer and ancillary data In the MEDiterranean sea (CARIMED).

OCADS excels in this arena because:

1. It is backed with a state-of-the-art long-term archive. All data are guaranteed to be available for at least 75 years, thanks to the NOAA records management requirements.
2. It has a stringent version control mechanism that ensures permanent preservation of all historical versions.
3. OCADS provides a community-driven metadata display interface that is tailored to the needs and preferences of the oceanographic research community.
4. Controlled vocabularies are applied to all aspects of its data management to ensure accurate data findability.
5. OCADS manages a wide range of ocean carbon and acidification data, including chemical, physical, and biological observations, as well as laboratory experiment results, and model outputs.
6. OCADS hosts one of the largest ocean carbon and acidification data repositories in the world, thanks to the data holdings transferred from CDIAC-Oceans.
7. OCADS has established mechanisms to support existing ocean carbon and acidification data product developments, e.g., SOCAT, and GLODAP.

## Results

### Mission

At its core, the mission of OCADS is to provide data management services that facilitate and support research on ocean carbon cycling and ocean acidification. This is accomplished through:

- Safeguarding their data in a well-supported federal archive to ensure long-term (≥75 years) access and version control,
- Serving as one of the world’s leading providers of ocean carbon and acidification data, information, and products, and
- Providing data management support for quality control, synthesis, and data product development activities.

The OCADS data management services include dedicated support for acquiring data, publishing data into the archive, managing rich metadata with controlled vocabularies, and enabling online data retrieval and access. Another aspect of the OCADS portfolio is QCing data and developing coastal and open ocean data products^23,49,50^. In addition, OCADS has been playing a leading role in establishing numerous international standards for both metadata and data^46,51^.

### Data scope

OCADS manages a wide range of ocean carbon and acidification data (Table 1). Here, ocean carbon and acidification data are defined as data that contain at least one of these variables: carbon dioxide molecular ratio (*x*CO_2_), pressure (*p*CO_2_) or fugacity (*f*CO_2_), total dissolved inorganic carbon content (DIC), total alkalinity content (TA), pH, hydrogen ion content ([H^+^]), carbonate ion content ([CO_3_^2−^]), and calcium carbonate mineral saturation states for aragonite (Ω_arag_) and calcite (Ω_cal_). Besides these variables, OCADS also serves as a repository for other Global Ocean Observing System (GOOS) biogeochemistry Essential Ocean Variables (EOVs), i.e., oxygen, nutrients (e.g., silicate, phosphate, nitrate), transient tracers (e.g., chlorofluorocarbons, or CFCs), nitrous oxide, particulate matter, stable carbon isotopes, and dissolved organic carbon^52^. Furthermore, OCADS is upgrading its infrastructure to support the management of mCDR data.

**Table 1:** An inventory of the OCADS data holding by observation type as of January 15, 2023.

Observation type	Number of datasets
Profile/CTD	1173
Surface underway	822
Time-series	101
Laboratory experiment	29
Total	2230

Common types of data OCADS manages include:

- *In situ* observational ocean carbon and acidification data, including chemical, physical, and biological observations collected from research vessels, ships of opportunity, moorings, and other uncrewed platforms.
- Results from physiological response studies, including laboratory experiments, mesocosm studies, field experiments, and natural analogues.
- Model outputs.
- Data products (See Table 2 for examples)

**Table 2:** Major regional and global ocean carbon and acidification data product development efforts, with OCADS providing data management support.

Data product	Abbreviation	Area	Citation
The Surface Ocean CO_2_ Atlas	SOCAT	Global	Bakker *et al*.^47^
The Global Ocean Data Analysis Project Version 2	GLODAPv2	Global	Lauvset *et al*.^48^
Coastal Ocean Data Analysis Product in North America	CODAP-NA	Coastal	Jiang *et al*.^49^
CARbon, tracer and ancillary data In the MEDiterranean sea	CARIMED	Regional	Álvarez *et al*.

### Product development support

OCADS provides data management support for numerous ongoing projects focused on developing ocean carbon and acidification data products (Table 2). This service comprises archiving individual datasets from various cruises, producing tables with comprehensive lists of these datasets, and providing access to the developed data products. At present, OCADS can only provide support for data product development activities related to ocean carbon and acidification research due to limited resources.

SOCAT is a global data product that provides surface ocean *f*CO_2_ measurements, primarily obtained from ships of opportunity (SOOP)^47^. It represents one of the most extensive collections of observational ocean carbon data. The latest release (SOCATv2022) contains 33.7 million *f*CO_2_ values with an accuracy of better than 5 μatm. A further 6.4 million *f*CO_2_ sensor data with an estimated accuracy of 5–10 μatm are available separately. GLODAP is a full water column open ocean data product, containing high-quality data from discrete bottle based measurements^48^. GLODAP covers 14 oceanographic variables, i.e., temperature, salinity, oxygen, nitrate, silicate, phosphate, DIC, TA, pH, chlorofluorocarbon (CFC-11), CFC-12, CFC-113, carbon tetrachloride (CCl_4_), and sulfur hexafluoride (SF_6_). The most recent release, GLODAPv2.2022, includes measurements from close to 1.4 million water samples collected on 1085 cruises. CODAP-NA is an internally consistent data product for discrete inorganic carbon, oxygen, and nutrients on the North American ocean margins^49^. CODAP-NA’s initial release (v2021) contains 3391 oceanographic profiles from 61 research cruises covering all continental shelves of North America, from Alaska to Mexico in the west and from Canada to the Caribbean in the east from 6 December 2003 to 22 November 2018.

### Best practice data standards

Common data standards are crucial in facilitating future data utilization, particularly for quality control and synthesis efforts. Adhering to such standards can minimize uncertainties and errors that may result from ambiguous variable abbreviations, inconsistent quality control flags, and non-standardized calculations. A recently released best practice data standard for discrete chemical oceanographic observations^51^ provides guidelines on various topics such as column header abbreviations, quality control flags, missing value indicators, standardized calculation methods for carbon system parameters, new tools for calculating thermodynamic variables using the International Thermodynamic Equations of Seawater 2010 (TEOS-10) equations^53^, and new tools for calculating *f*CO_2_ from dry-air mixing ratios.

### Components

**Fig. 2:**
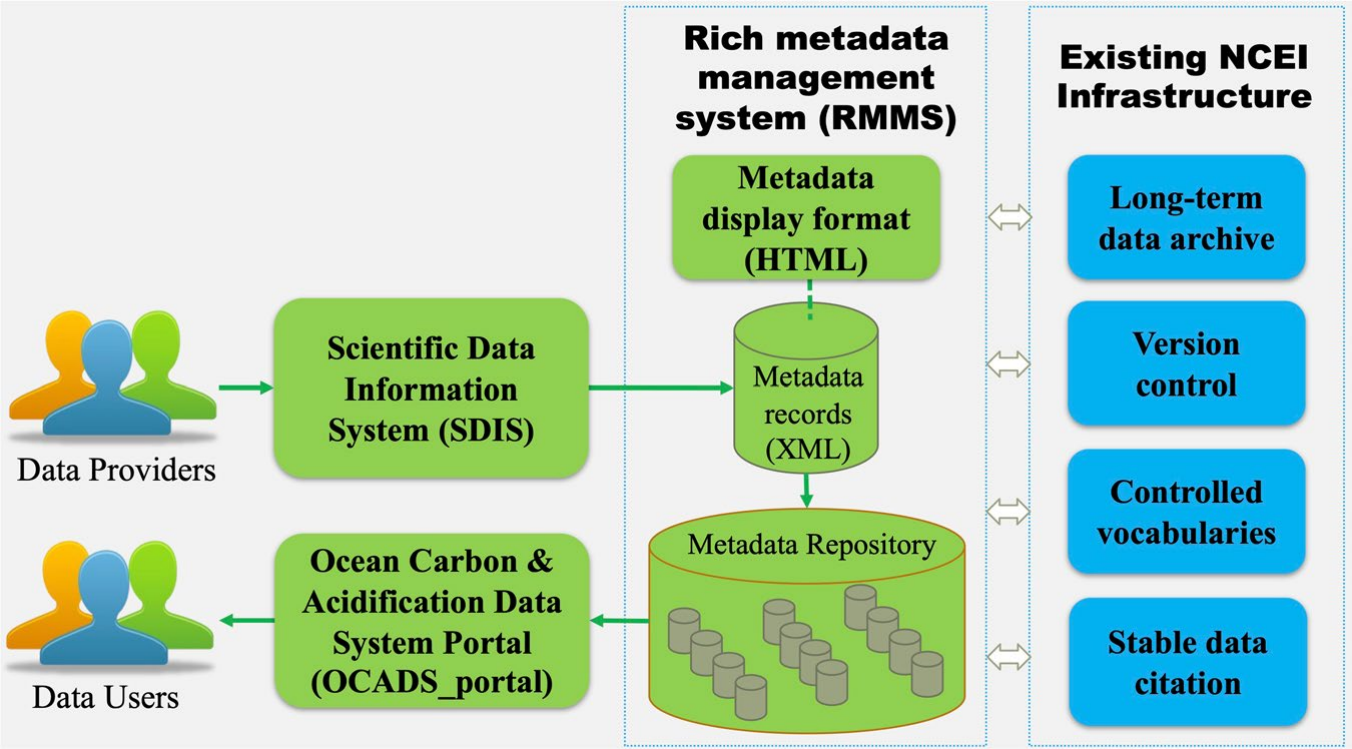
A schematic diagram showing the major components of the Ocean Carbon and Acidification Data System (OCADS).

OCADS comprises three primary user interfaces that have been tailored to provide optimal data management support for research related to ocean carbon and acidification (Fig. 2):

- The Scientific Data Information System (SDIS), a digital data submission interface,
- The Rich Metadata Management System (RMMS), a user-friendly metadata management and display interface, and
- The Ocean Carbon and Acidification Data System Portal (OCADS_portal), a data search and access interface.

**Fig. 3:**
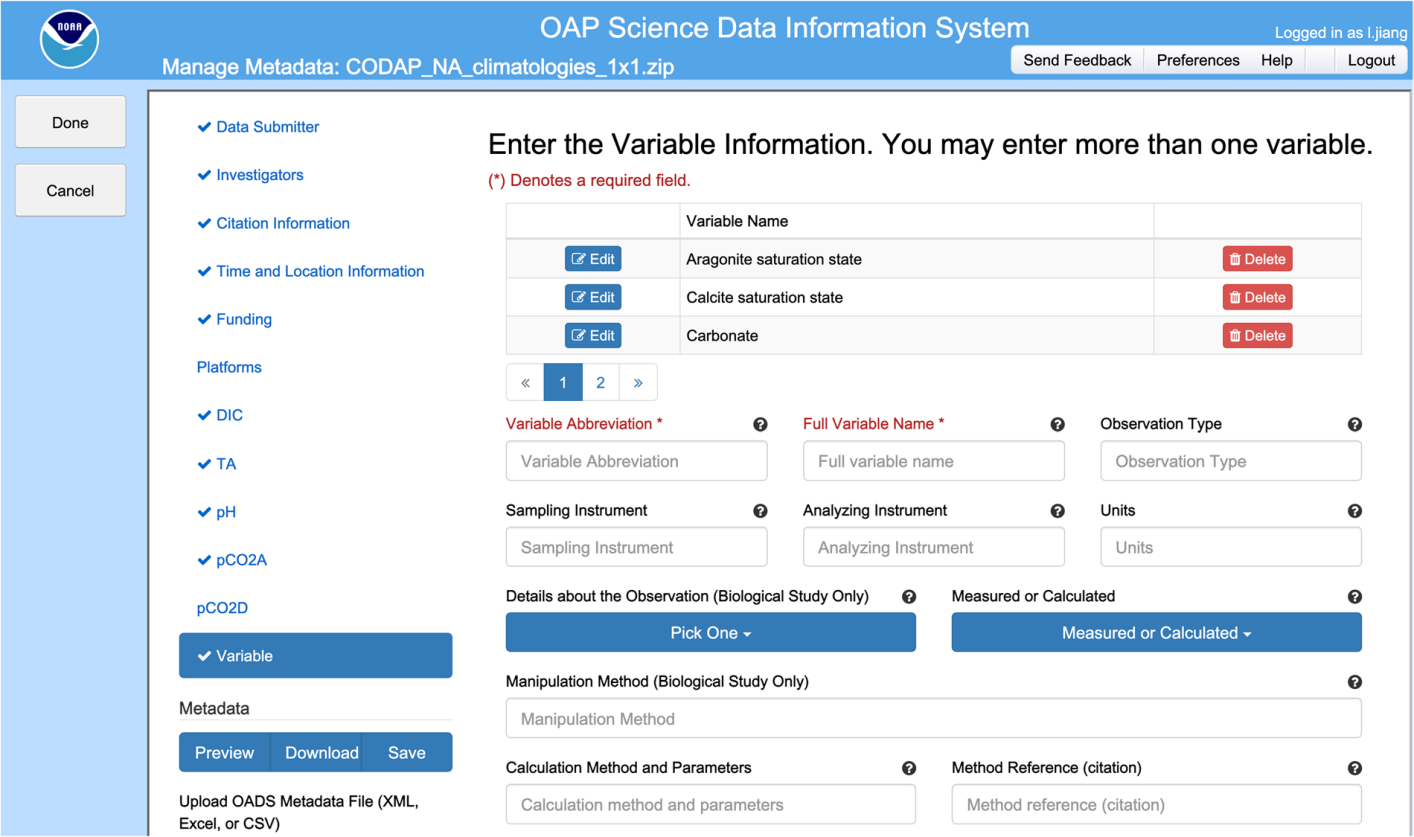
A screenshot showing the metadata interface of the Scientific Data Information System (SDIS, https://data.pmel.noaa.gov/sdig/oap/Dashboard/).

The **Scientific Data Information System (SDIS)** is a digital data submission interface developed by NOAA’s Pacific Marine Environmental Laboratory (PMEL) to streamline the process of submitting ocean carbon and acidification data to NCEI. (Fig. 3). It enables a user to input metadata, upload data files, and submit the resultant data package to NCEI for review and eventual archival. The SDIS integrates rich metadata management capabilities that are designed to satisfy the needs and requirements of the global ocean carbon and acidification research community^46^. The SDIS incorporates a user profile management system that permits data submitters to (a) maintain a record of all their prior submissions, (b) start a new submission by duplicating an existing record, and (c) save their work midway through a submission and resume later. In addition to new submissions, the SDIS also supports revision requests.

The **Rich Metadata Management System (RMMS)** is a metadata management and display interface that has been created to present the collected rich metadata information in a user-friendly format. At present, the RMMS is comprised of these sections: title, investigators, package description, data citation, identification information, types of study, a browse graphic, temporal coverage, spatial coverage, platforms, research projects, variable metadata sections, datasets related to this current dataset, and funding information^46^. The RMMS plays a crucial role in providing data management experiences that meet the needs and preferences of the global oceanographic research community.

**Fig. 4:**
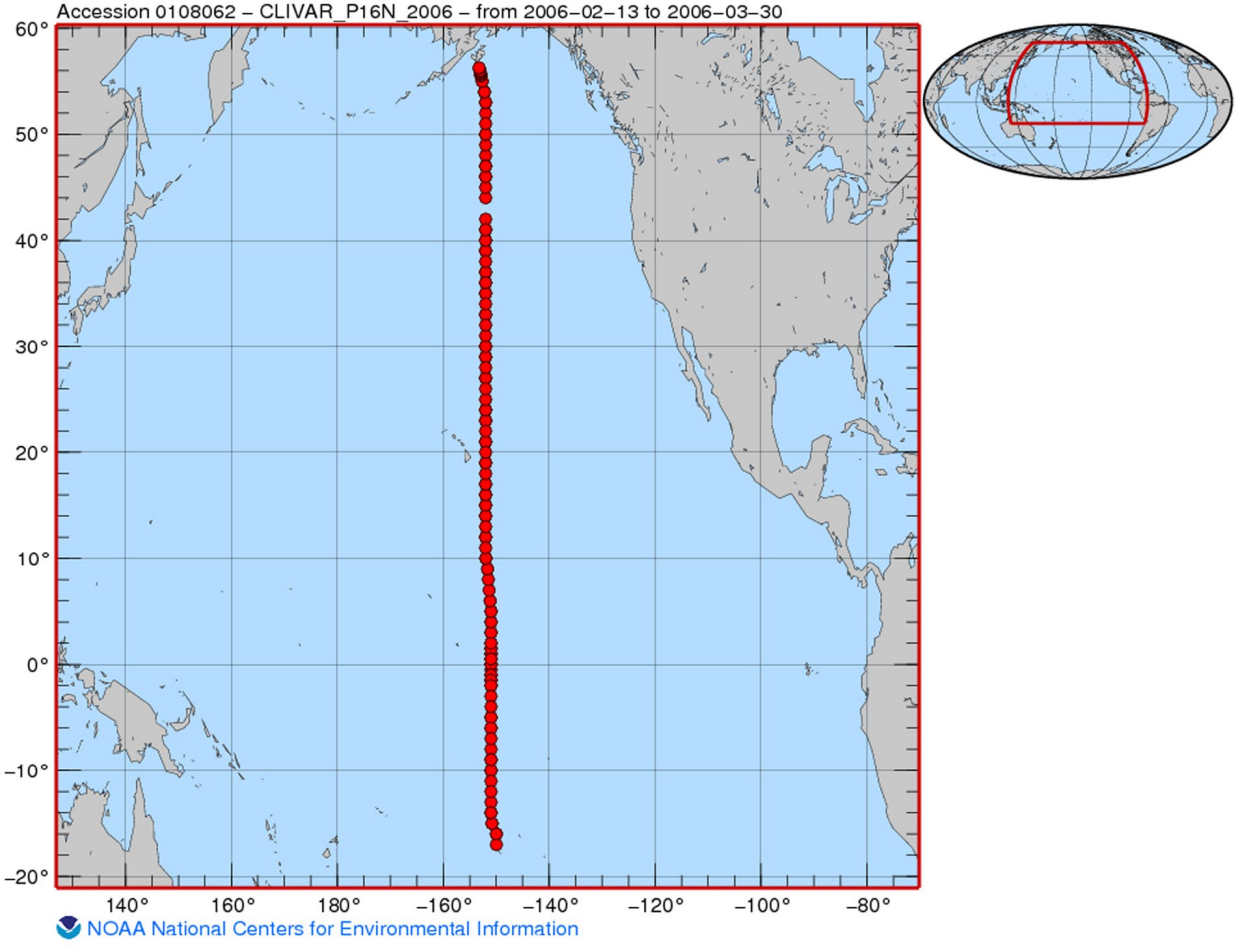
An example of a browse graphic from a dataset collected in the Pacific Ocean from 2006-02-13 to 2006-03-30 (Cruise_ID: CLIVAR_P16N_2006) (Credit: https://www.ncei.noaa.gov/data/oceans/ncei/ocads/metadata/0108062.html).

To ensure uniformity and ease of understanding, all OCADS dataset titles adhere to the template of “[observed properties] collected from [observation categories] using [instruments] from [research vessels or other platforms] in [sea names] during [research projects] from [start date] to [end date]. An example of an OCADS data title is: “*Dissolved inorganic carbon, total alkalinity, pH, temperature, salinity and other variables collected from profile and discrete sample observations using CTD, Niskin bottle, and other instruments from R/V Wecoma in the U.S. West Coast California Current System during the 2011 West Coast Ocean Acidification Cruise (WCOA2011) from 2011-08-12 to 2011-08-30*”. Each OCADS dataset is accompanied by a browse graphic, providing users with a visual representation of the sampling locations or experiment setup, facilitating a quick understanding of the data (Fig. 4).

**Fig. 5:**
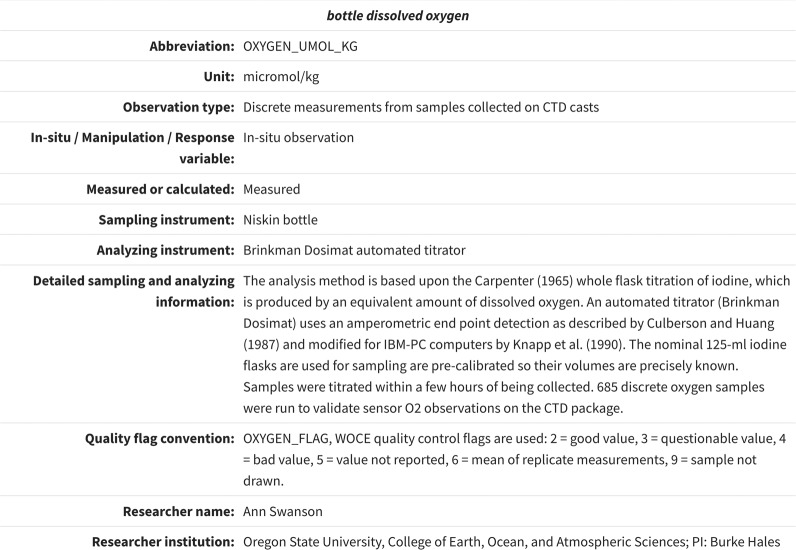
An example of a “variable metadata section”^46^ from a dataset during the 2011 West Coast Ocean Acidification Cruise (Credit: https://www.ncei.noaa.gov/data/oceans/ncei/ocads/metadata/0123467.html).

The “variable metadata section” is a one of the most important components of the RMMS, as it allows for the detailed documentation of all ancillary information related to a particular observed property, e.g., dissolved oxygen. This section can be repeated as many times as needed to document rich metadata information for all observed properties in the dataset. Information contained in this section will assist data users in comprehending the measurement details, such as the instruments used, methods applied, calibration procedures, and the associated data quality and uncertainty (accuracy/precision). Accuracy refers to how close the measurements are to the true or known values. Precision, on the other hand, refers to how close the measurements are to each other. Future improvements include the inclusion of a new metadata field that specifies whether a measurement is of weather or climate quality^54^, as well as additional elements aimed at supporting mCDR research, e.g., types of alkalinization. An illustration of a variable metadata section is presented in Fig. 5. Note that only the metadata elements that have been filled out by the data producers are shown, although the RMMS is capable of  displaying all metadata elements as described in Table 2 of Jiang *et al*.^46^.

**Fig. 6:**
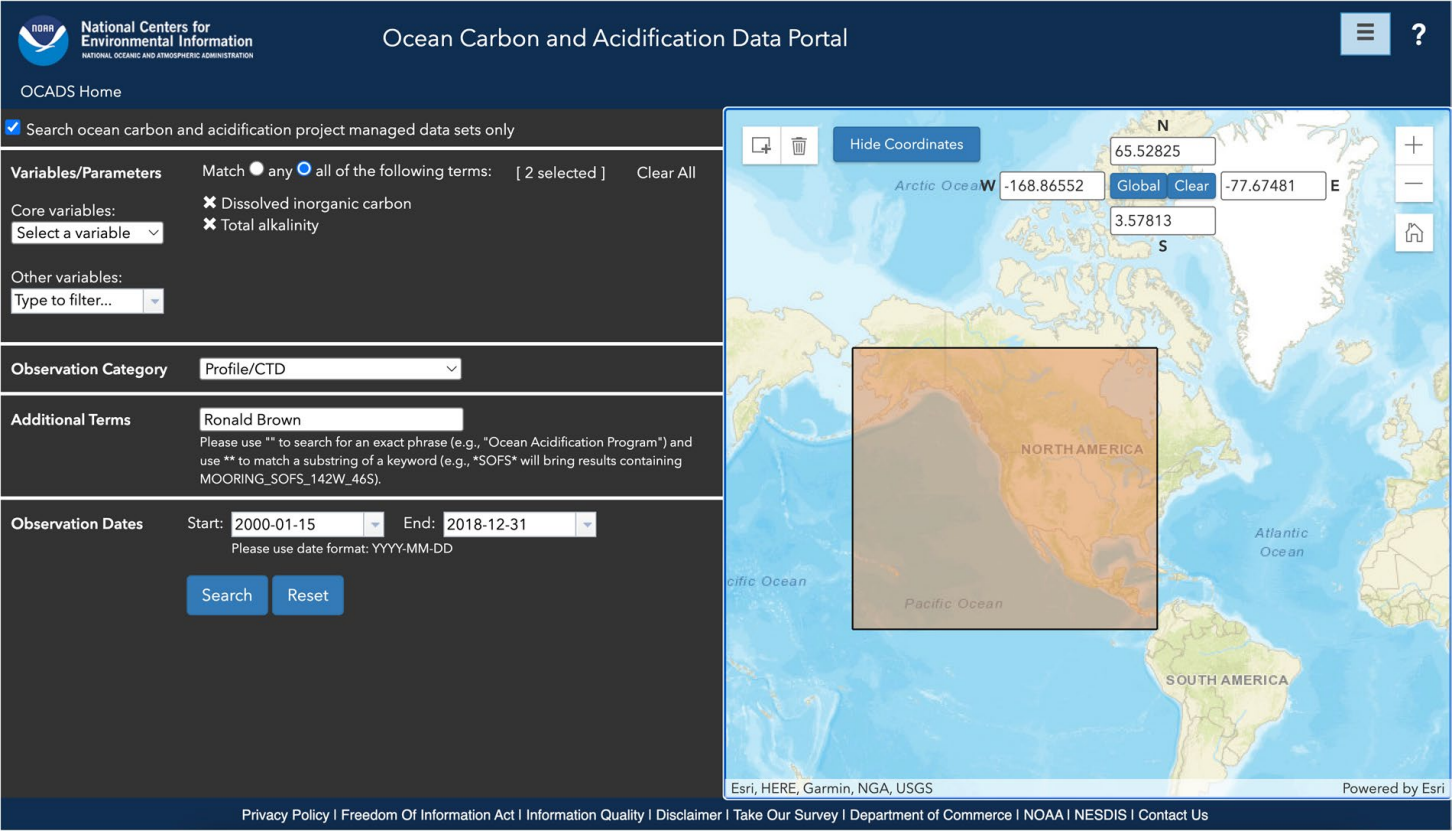
A screenshot showing the Ocean Carbon and Acidification Data System Portal (or OCADS_portal, https://www.ncei.noaa.gov/access/ocean-carbon-acidification-data-system-portal/).

The **Ocean Carbon and Acidification Data System Portal (OCADS_portal)** provides an interface that enables users to search for and access all of OCADS’ data holdings (Fig. 6). It utilizes user-friendly technologies, such as auto-complete, dropdown menus, and OpenLayers maps, to enhance the user experience of searching for data. Currently, the Portal enables users to search for datasets based on five criteria:

- **Variables/Parameters:** The observed properties, e.g., water temperature, total alkalinity content, etc. A user can select one or multiple observed properties.
- **Observation Category:** How the observed properties are measured, e.g., surface underway, time-series, CTD profile, laboratory experiment, etc.
- **Additional Terms:** A powerful free-text search box allowing users to use any other elements of the metadata record to assist with the search, e.g., a research vessel name, the last name of an investigator, an expedition code (EXPOCODE), a cruise identifier (Cruise_ID), etc^51^.
- **Observation Dates:** The start and end dates of the observation. Only cruises with at least one data point collected during the period will show up in the search results.
- **Spatial Coverage:** A user can either input the bounding box information (longitudes and latitudes) or draw a rectangle on the map to define the spatial constraints of the query. Similarly, only cruises with at least one sampling station within the bounding box will show up in the search results.

### Customers

OCADS recognizes the significance of (a) documenting all ocean carbon and acidification data with common metadata and data standards and controlled vocabularies, and (b) making them available through a single data portal. The program encourages data submissions from around the world and provides this service at no cost to all data producers, regardless of their location. In addition to data producers, OCADS also serves data users and consumers, including researchers, educators, decision-makers, private industry, and the general public from all nations.

### Helpful tips

Here are some additional tips that will help users access data at NCEI:

- An “accession” refers to a dataset published at NCEI’s archives. The accession number is a 7-digit numerical number used to uniquely identify a dataset archived at NCEI.
- A “landing page” refers to NCEI’s generic metadata page, in contrast to the RMMS page that is served through OCADS. The former covers bare minimum elements such as title, abstract, citation, and keywords. The latter is much richer and is focused on providing the best metadata to meet the data needs of ocean carbon and acidification research.
- If only an accession number is available, below is how to access its metadata:
  - The RMMS page (available only to datasets published through OCADS, NNNNNNN is the accession number): https://www.ncei.noaa.gov/data/oceans/ncei/ocads/metadata/NNNNNNN.html
  - The landing page (available to all datasets within NCEI’s archives): https://accession.nodc.noaa.gov/NNNNNNN
- If a user is taken to the landing page first by search engines, here is how to access the RMMS page: first click the “Documentation” tab, then follow the “Project metadata” link.
- To see the version history of an accession: first go to the landing page, then click the “Lineage” tab.

### Links & Email

- Ocean Carbon and Acidification Data System (OCADS):
  - https://www.ncei.noaa.gov/products/ocean-carbon-acidification-data-system/.
- Scientific Data Information System (SDIS):
  - Link: https://data.pmel.noaa.gov/sdig/oap/Dashboard/.
  - Video tutorial: https://www.youtube.com/watch?v=ZZL_wQWr38A.
- Rich Metadata Management System (RMMS):
  - An example: https://www.ncei.noaa.gov/data/oceans/ncei/ocads/metadata/0123467.html
- Ocean Carbon and Acidification Data System Portal (OCADS_portal):
  - Link: https://www.ncei.noaa.gov/access/ocean-carbon-acidification-data-system-portal/.
  - Video tutorial: https://www.youtube.com/watch?v=DYFI0aH00FU.
- OCADS contact:
  - Email: noaa.ocads@noaa.gov.

## Discussion

### OCADS and international OA data management

OCADS operates within the Oceanographic Science and Development Branch (OSDB) of NCEI. As a component of the federal government, OCADS enjoys stable funding, which sustains its data management system. However, this does not imply that OCADS is biased towards NOAA or the United States. NCEI, the parent organization of OCADS, is a World Data Service for Oceanography (WDS-Oceanography) designated by the International Council for Science (ICSU) through a resolution of the 29th United Nations General Assembly in 2008^55^. As with all data handled by NCEI, 100% of the data obtained by OCADS will be made publicly available to all users worldwide.

OCADS recognizes the importance of collaborating with other Data Analysis Centers (DACs) to achieve the common goal of offering top-notch data management services to the global oceanographic research community. Such partnerships not only help distribute the burden of international ocean carbon and acidification data management efforts, but also help overcome any regional or geopolitical barriers that could potentially prevent direct interaction between OCADS and local data producers. For DACs that need the support of a long-term archive, OCADS is eager to cooperate with them to transfer a copy of their data to NCEI’s archive. This process is generally automated.

Ideally, all DACs, including OCADS, should function as regional nodes, aiding in the availability of all ocean carbon and acidification data via a centralized one-stop data portal. This can be accomplished by providing standardized metadata to the search engine of the agreed-upon one-stop portal. The most recent U.N. Ocean Acidification Research for Sustainability (OARS) data management initiative suggests designating the Global Ocean Acidification Observing Network (GOA-ON) Portal as the envisioned one-stop OA data portal. Once this is implemented, users can employ the GOA-ON Portal to search for and retrieve all ocean carbon and acidification data of a specific type. After finding a dataset through the Portal, a user can be directed back to the respective regional DAC to access the data files and locate any pertinent metadata information.

For such a federated system to work properly, the participating DACs must fulfill the following minimum requirements:

1. Maintain a long-term archive to ensure uninterrupted data access into the future.
2. Provide strict version control capabilities, preserving all historical versions of a dataset on a permanent basis.
3. Utilize a community-driven common metadata template, e.g., the one used by OCADS, to collect comprehensive metadata information needed for ocean carbon and acidification research.
4. Present the collected metadata in:
  - A user-friendly interface for metadata readability (e.g., HTML).
  - A technical format to facilitate machine-to-machine interoperability (e.g., XML, SQL).
5. Employ common controlled vocabularies for successful data findability and easy machine-to-machine metadata exchange.
6. Support data citation with DOIs.
7. Establish a mechanism for sharing metadata with the agreed-upon one-stop portal, making the data searchable and accessible through the portal.

Before such a federated system is implemented, it is recommended that data producers share a copy of their data with OCADS to ensure timely inclusion in data products like SOCAT and GLODAP.

### Data management vs. data product development

The concepts of “data management” and “data product development” are often conflated, but they are fundamentally different. It is true that they both involve working with data, but their similarities end there. Data management refers to the process of ingesting, storing, and disseminating data^43^. It includes tasks such as establishing long-term archives, maintaining version control, using metadata templates, adhering to data standards, and implementing controlled vocabularies. Data management is frequently mandated by government regulations and carried out by specialized national data centers.

Data product development, on the other hand, refers to the process of developing products and services (e.g., synthesis products, gridded climatologies, models, or predictions) out of data for the purpose of providing value to users. Unlike data management, data product development is rarely mandated by federal or local regulations. It can be carried out by anyone, including academic institutions. Like other research activities, data product development also needs data management support.

## Methods

OCADS was designed and built with the following rationales in mind:

- **A customer-driven service:** OCADS was designed to meet the data management needs and requirements of the global ocean carbon and acidification research community. We believe data management is a scientific effort that requires close collaboration with the research community. To ensure that our service is customer-driven, we rely on researchers to help us define many aspects of the OCADS data management, including metadata templates, controlled vocabularies, data standards, website design, and data submission and access interfaces.
- **Rich metadata management:** OCADS utilizes a community-driven rich metadata template^46^ to collect detailed metadata information to enable data users, especially the synthesis community, to comprehend the measurement uncertainty and other sampling and calibration details, e.g., instrumentation, calibration, scales, units, biological subject, life stage, etc. of each variable. Rich metadata management is applied to the submission interface, metadata display interface, and the data search and access interface.
- **Controlled vocabularies:** Controlled vocabularies are lists of standardized terms. For example, some scientists could report their dissolved inorganic carbon measurements as “total dissolved inorganic carbon content”, while others may call it “dissolved inorganic carbon”, or “total carbon dioxide”. Multiple variations of terms for the same variable may cause confusion or decreased findability. Therefore, controlled vocabularies play a very important role in ensuring successful data search. At OCADS, controlled vocabularies are applied to many groups of metadata elements, including observed properties (e.g., dissolved oxygen), observation types (e.g., surface underway, time-series), platforms (e.g., research vessels), sea names, instruments, institutions, people, countries, etc.
- **Stable data citation:** Each dataset archived at NCEI has an associated data citation. The citation contains information such as author list, title of the data, data repository, publication year, and an optional persistent identifier (e.g., DOI). DOI is strongly recommended for all OCADS datasets, although OCADS gives data producers  the discretion of whether a DOI should be assigned or not. According to the NOAA Plan for increasing Public Access to Research Results (PARR)^56^, “*NOAA National Data Centers are the only entities authorized to issue NOAA dataset identifiers*”. This important guideline ensures that the same dataset will not be assigned multiple DOIs, potentially causing unnecessary confusion.
- **Long-term archive:** All OCADS datasets are archived using the NCEI enterprise archival infrastructure, ensuring the data will be available into the future (≥75 years). Besides a digital copy at NCEI’s server, all published datasets in NCEI’s archives are backed up to a staging disk on a backup server, as well as two offline tape-based repositories. Any data that are either new or changed are first backed up to the staging disk at a frequency of once per day, 7 days per week. Data on the staging disk is then migrated to tape copy #1 (on-premises). The same data are later copied to tape copy #2 (off-site).
- **Version control:** Unlike journal publications, datasets are often updated after they are published. This can happen after further QC is conducted or after additional data or metadata are gathered. While it is critical to provide future users with the latest version of the data, it is equally important to preserve all historical versions. Otherwise, research based on historical versions of the data can no longer be verified. In rare cases, the data may need to be reverted to a previous version. NCEI’s enterprise archival infrastructure provides strict version control. After a dataset is published, no further changes can be made to the published version. Any changes to the data will trigger a new version of the dataset.
- **Preserving the original data:** In an era when the Findable, Accessible, Interoperable, and Reusable (FAIR) Data Principles^57^ are often emphasized over traditional data formats like Microsoft Excel, OCADS recognizes the importance of preserving the original data. They often contain embedded QC comments, equations between different columns, color-coding, etc., which are critical to the future data use. Such information can sometimes get lost during the conversion to FAIR-compatible data formats, e.g., NetCDF. Therefore, OCADS always ensures the preservation of the original data.

## Data Availability

All data presented in this article are available at https://www.ncei.noaa.gov/products/ocean-carbon-acidification-data-system/.
